# Pulp-Stones: Their Significance, Methods of Diagnosis, and Necessary Treatment

**Published:** 1892-04

**Authors:** Rodriguez Ottolengui

**Affiliations:** New York


### PULP-STONES: THEIR SIGNIFICANCE, METHODS OF DIAGNOSIS, AND NECESSARY TREATMENT.[1]

BY DR. RODRIGUEZ OTTOLENGUI, NEW YORK.

What is a pulp-stone? The name is applied to globular masses found either in the body of the pulp tissue or attached by a pedicle to canal walls. They are seen by microscopic examination to be of at least three varieties,—one having the appearance of secondary dentine, one presenting with true bone lamellæ, and a third being a combination of bone and dentine. The last is the rarest. It is not my purpose to discuss the histological aspect of this subject, the time allowed me by your committee for the preparation of this paper being too brief. I have, however, within my own experience, been fortunate, or unfortunate, enough to meet with many instances where this disturbance was the exciting cause of pain, and I have collated facts which may be of clinical value. These I shall now present to you for your consideration.

I shall begin by relating a few typical cases from practice, from which I may be able to draw some deductions.

Case I.—Mrs. A., aged about twenty-six, married, no children. This patient was of phlegmatic temperament, cleanly habit, and in good health. Nevertheless, her teeth were blue-white, deficient in lime salts, excessively sensitive, and when caries occurred the destruction was usually rapid. Presented with a leaky amalgam filling in anterior proximate surface of superior first molar. This was removed, and some softened dentine found throughout the cavity. Upon attempting to remove this, the tooth was found to be very sensitive. The cavity was, notwithstanding, thoroughly cleansed,

---

[1] Read before the Central Dental Association of Northern New Jersey, May 18, 1891.

the pulp was not exposed, but nearly approached, and because of the sensitiveness of the tooth it was filled with a probationary filling of oxyphosphate. The patient returned in two days, complaining that the pain which first brought her to the office had not been alleviated. Filling was removed and a minute examination made for a possible exposure, none being found. Was dressed with equal parts of oil of cloves and carbolic acid, covered by gutta-percha. Returned on the following day, and for two succeeding days, upon each of which the treatment was renewed. The history was that for a number of hours after each dressing the pain would be in abeyance, but upon assuming the recumbent position at night it would ensue with increasing severity. From loss of sleep and persistent pain, by this time the patient was growing excessively nervous and desirous of having the tooth extracted. I therefore decided to apply arsenic, and used the paste supplied by the depot. This application was made at ten in the morning. Patient returned at two in the afternoon, reporting that the pain was unendurable. Dressings were removed and various medicaments essayed to allay the pain, none of which produced any effect. Gave patient ether, not sufficient to produce complete anæsthesia, and natural sleep followed, which continued for three hours, when she awakened free from pain. She was dismissed for the night with a light anodyne dressing in the cavity. The pain recurred about two in the morning and banished sleep. At nine in the morning she reported, and I succeeded in removing only a slight portion of the dentine covering the pulp, when I was stopped by the extreme sensitiveness. I renewed the arsenical dressing and the experience of the previous day was repeated. It must be remembered that up to this time I had never seen a pulp-stone, and was entirely ignorant of diagnostic symptoms. To me there seemed nothing to do but to pursue the arsenical treatment till the sensitiveness should be controlled, and it was a mystery to me that such resistance should be possible. This treatment was continued from day to day, until at the end of four days I was enabled to cut away the dentine down to a hard surface which resisted an ordinary engine bur. It appeared of a greenish color, and was as large in circumference as the end of a small lead-pencil. This I drilled around and finally dislodged so that I lifted it out with an excavator. I found it to be a pulp-stone which entirely filled the pulp-chamber, and attached to it was the pulp from the palatal root. This pulp was stiff enough to stand erect when the pulp-stone was laid on the bracket, and when rubbed between the fingers felt gritty. The buccal pulps bled freely, and could not be removed without further arsenical treatment. After their removal they were found not to exhibit any granulation that could be determined by the fingers, and undoubtedly they were the seat of the pain. The tooth was filled and no further trouble ensued.

Case II.—Miss R., a young woman, unmarried, aged about twenty, excessively nervous temperament, reported with toothache in a lateral incisor. Found a small opening into which a fine broach could be forced, causing pain, but no hemorrhage. Applied arsenic, and patient returned in two hours in torture. Anodyne dressings relieved the pain, and she was dismissed for the day with no further application of arsenic. On the next day the arsenic was renewed, and the unpleasant symptoms of intense pain ensued, necessitating removal as before. This was repeated for four days before the pulp could be taken out, when it came away in a single bloodless mass, appearing like a grain of rice, and nearly as stiff, being readily broken into distinct fragments. Strictly speaking, I suppose this was calcification of the pulp rather than true pulp-stone.

Several instances of this nature awakened me to the fact that persistent resistance to arsenic is a good diagnostic symptom of the presence of pulp-stones. I removed, from some cases, several stones before reaching a portion of the pulp which would bleed.

Case III.—Mrs. S., a stout married woman, very nervous temperament, aged about forty, reported that she had been in the hands of her family dentist who had treated with arsenic six times, resulting in torture, and no effect whatever upon the sensitiveness of the cavity. She presented with a note from her dentist asking me for advice and assistance, as he was mystified. From the similar experiences which I had had, I determined at once that pulp-stones were present, and considered this an opportunity to attempt a new treatment which I had decided should be essayed at the first chance. I administered nitrous oxide and kept the patient under its influence until I had drilled around and removed the pulp-stones. This was a lower second molar, and it was necessary to renew the gas as often as she appeared about to awaken before I could gain sufficient time to dislodge the pulp-stones during anæsthesia. I took out, as usual, first, one large stone, which covered the top of the chamber, and then found the chamber occupied with four others, a fifth and sixth covering the entrances to the canals. As soon as I had reached the soft tissue, I allowed the patient to awaken, when, to my disgust, I found the remainder of the pulp too sensitive to be removed. I dressed with oil of cloves and carbolic acid, and the following day reamed out both canals, the patient being once more put under the influence of the gas.

Case IV. brings us to another feature of this disturbance. Mr. B., a man of thirty, presented with a superior second bicuspid, cavity deep and sensitive, but pulp not exposed. I filled with oxyphosphate as a permanent filling. Patient returned two years later reporting intense pain. The tooth being of good color, and not responding to any of the investigations in search of signs of pericementitis, the case was diagnosticated as pulpitis, due to the fact that possibly a layer of dentine which was carious had been left as a cap for the pulp, and that this caries had continued until it reached the pulp, causing the irritation. I will say, parenthetically, that this has never been my practice, but in this instance I supposed it might have occurred unintentionally. I removed the filling, and found the cavity walls hard and entirely free from caries. The pulp was not exposed, and the tooth was sensitive to the excavator. I dressed with oil of cloves and dismissed. The patient, however, returned the same afternoon, reporting intense pain. Though this was the first of this class of cases that had come to me, I diagnosticated pulp-stones, and proceeded upon my usual method. I was afraid to apply arsenic at the afternoon hour, because my patient could not reach me again that night. I determined, therefore, to extirpate the pulp under nitrous oxide, and did so, removing three pulp-stones from the entrance to the canal, and the remainder of the pulp on a broach.

Case V. was somewhat similar, but presented with most disagreeable after-results. The patient was a married woman, about thirty-five, and in good health. I found a badly-leaking gold filling in the lower second molar, involving the posterior proximate and crown surfaces. Upon removing the gold I found a phosphate capping in good order. Not deeming it a favorable position for gold, I filled with amalgam, and on the following day polished thoroughly at all points. In three days patient reported in pain, and after this had continued for three days more, I removed my filling and also the capping, whereupon the pulp bled freely and the pain subsided. I applied arsenic, not suspecting pulp-stones for an instant, because the surface of the pulp presented soft tissue. A night of agony followed, and patient came early on the following day in a nervous, weakened condition. I attempted to remove the pulp, and, though causing pain, did extirpate it from the pulp-chamber. Upon close examination, rubbing it between the fingers, I found entangled within its meshes five small globular masses which, when washed in water, presented the usual appearance of pulp-stones. Exploration disclosed the fact that the pulp-canals were confluent, and the rest of the pulp was removed with a Gates Glidden drill, which, however, did not enlarge the canal. As there was a considerable hemorrhage, I dressed the canal with tannin and glycerin, covered with gutta-percha. The patient had a very bad night, and many more thereafter. It was two weeks before the tooth showed any signs of restoration to a comfortable condition, which only ensued after I changed the dressing to one of aconite and iodine, essential oils having been my first reliance.

Case VI. is very similar in its history to the last, and is introduced because of the large number of stones found. This was a married woman of forty, and the tooth also a second lower molar, the cavity occupying the anterior and crown surfaces. The tooth was filled with phosphate by her previous dentist, but for over a year had never been entirely comfortable. I removed the phosphate, and the pulp bled. I applied arsenic, which caused a night of wakefulness, and the following day I removed seventeen pulp-stones, all small, but distinct globules, the rest of the pulp being one mass of granular bodies interwoven with the tissues. Having reamed out both canals as thoroughly as possible, I came to the conclusion that the roots curved at their extremities so that a drill could not reach their foramina. With the dam in place, however, I did reach the ends by use of the Evans root-dryer. The canals, being thoroughly dried, were soaked with oil of cloves, dried out and soaked again, and then a dressing applied, which I left in for twenty-four hours, upon which I filled the root-canals with gutta-percha. About this time sickness occurred in the family, and I did not see the patient again for two weeks, when she reported that she had suffered pain. I therefore removed the filling in the roots and applied dressings for three days, whereupon I again filled them. As the cavity was a very large one, and the patient, with another similar cavity, had suffered much from the tediousness of prolonged filling, I determined to fill with gold at two separate sittings. At the time when I filled the roots, I also filled the tooth half full with gold and polished the proximate surface, intending to insert the rest of the gold as a distinct filling. The following day the patient came in accompanied by her husband, reporting that she had been compelled to take morphine injections the night previous. I was lucky enough to be able to reach both canals and remove filling without disturbing the bulk of my gold. Relief followed, but it was two weeks before I dared to refill the roots, an abscess having developed, and even then I filled with cotton and oil of cloves, covering with gutta-percha, which was left in place for four months before I filled permanently. Then the roots were filled with gutta-percha, and the gold filling completed.

I must report one more case as typical of another point which I wish to bring out.

Case VII.—A married woman, aged about thirty. A lower second molar; pulp exposed. I capped and filled with phosphate, gutta-percha being used for the cap. This phosphate lasted four years, when it became necessary to refill the tooth. At this time I found the pulp had recovered itself, and therefore I filled with gold. Six years afterwards, or ten years from the time when the pulp was capped, I was compelled to remove the tooth because of a painful abscess. Examination made immediately after the removal of the tooth showed both canals filled with pulp-stones till near the extremity, the lower portion of the pulps being putrescent, and undoubtedly the cause of the abscess.

These cases will suffice upon which to hang my arguments and deductions.

Exactly what the histological process is which produces these formations, whether the odontoblastic layer secretes the lime within the tissue, as is claimed, or whether we accept Bödecker's idea that the odontoblasts are but medullary corpuscles, and that it is possible for medullary corpuscles to appear within the body of the pulp, subsequently producing the depositions under the stimulus of pabulum supplied by the blood-vessels. I think we may adopt the following theories:

We may consider that the pulp of a human tooth is the residue of the dentinal germ. During fœtal life this germ followed its function and produced dentine around itself, contracting its own size by the process. I think there is abundant evidence to substantiate the claim that this action is continued after adult life, though of course much less rapidly. That is to say, the canals of teeth are constantly decreasing in diameter by deposition of dentine. Now, we know that in all embryonic tissues there resides a mysterious intelligence which directs its actions so as to produce a given form. In embryonic life there is a period when, from microscopic appearance, man cannot differentiate between bone tissue and muscle tissue. Nevertheless there is undoubtedly a difference, at least in the element of intelligence, since bone will be invariably formed of a given continuity, and muscles occur with exactness of relation and purpose. If we cut off the end of a finger, and nutrition is not impaired or disturbed, the inflammatory process which follows results in a return to embryonic conditions, succeeded by a rebuilding along the original lines, so that the finger-tip is precisely restored. Should there be a disturbance, however, between the relations of nutrition and organization, the result is a building of tissue, but it is unorganized, and the result is hypertrophy. This hypertrophy will be in line with the functional intent of the part, —that is, in bone we get an exostosis, while in the soft tissues we find a growth of soft tissue.

If we examine the pulp we find the conditions similar, but the environments are different. In the case of the finger-tip, nothing but the surrounding atmosphere interferes with the growth outward. In the bones, the exostosis is possible, because the surrounding tissues are soft and readily absorb before the growing bone. The pulp, however, is surrounded by bony walls, which do not yield readily, though Professor Peirce has just reported a case in which inflammation produced hypertrophy of the pulp and absorption of the canal walls to accommodate the increase in the size of the pulp. However, we may say that the action would be thus: If caries reaches the pulp, and does not cause its death, hypertrophy of the soft tissue will follow, the removal of the circumscribing wall at the point of caries allowing the pulp to grow outward, until we find it occasionally filling the cavity of decay. If, however, the inflammatory action is induced, perhaps by some micro-organism which has the power to penetrate dentine rapidly, before the pulp is actually uncovered two things may occur. The more likely result is the death of the pulp, caused by a supervening suppurative inflammation. If, however, the inflammatory action is dilatory, we can readily imagine a condition where the functionary activity being disturbed, the lime is deposited so rapidly that complete organization is prevented, and consequently we have a condition similar to that which would produce an exostosis in a bone. As the deposition, or bony growth, cannot be external, because of the limiting walls, the result is, the soft tissue of the pulp itself gives place to the bony masses, or dentinal masses, which we term pulp-stones.

If we accept this doctrine, we come inevitably to this conclusion: The sometime considered healthy action of a pulp in recovering itself is really a pathological action, and one which will not discontinue until the death of the pulp ensues, as in the last case reported; the canal is filled with pulp-stones until what is left of the pulp dies, when putrefaction, and subsequent abscess, is to be expected.

From the above I draw these conclusions:

*First.* Intense pain, without decrease of sensibility, following the use of arsenic, is a reasonable sign of the presence of pulp-stones.

*Second.* Intense pain following a probationary filling, whether immediately or after an extended period of time, there being sensibility in the dentine, and no symptoms indicating other disease, the pulp not being exposed, pulp-stones are to be suspected.

*Third.* Where it is suspected that pulp-stones are present, arsenic must not be used, and if it has been used once, it must not be repeated. The pulp must be extirpated under an anæsthetic.

*Fourth.* Where pulp-stones have been removed till soft tissue is reached, arsenic must not be used for controlling the sensibility of the remainder. It is preferable to use an anæsthetic.

*Fifth.* After the removal of pulp-stones, it is not safe to insert a permanent filling immediately; the canals may be filled, but should be covered with a readily removable probationary filling.

*Sixth.* If a pulp be exposed, capping must produce either its death or else set up an inflammatory action which will be followed by deposition of pulp-stones, which will ultimately strangle the pulp. It is therefore inexcusable to cap any pulp which has bled, especially in young patients.

